# Immune checkpoint inhibitor–related myocarditis in a patient with hepatocellular carcinoma: a case report

**DOI:** 10.3389/fcvm.2026.1749534

**Published:** 2026-06-03

**Authors:** Yuqiang Zhou, Zhiliang Zhang, Shasha Liu, Jiang Nan, Zhuoli Zhang, Zixian Chen

**Affiliations:** 1 The First School of Clinical Medicine, Lanzhou University, Lanzhou, China; 2 Department of Pathology, The First Hospital of Lanzhou University, Lanzhou, China; 3 Department of Radiology, The First Hospital of Lanzhou University, Lanzhou, China; 4 Departments of Radiological Sciences, University of California, Irvine, CA, United States

**Keywords:** cardiac magnetic resonance, endomyocardial biopsy, hepatocellular carcinoma, immune checkpoint inhibitors, immune-related adverse events, myocarditis

## Abstract

Immune checkpoint inhibitors (ICIs) are pivotal in treating advanced malignancies, including hepatocellular carcinoma (HCC). However, they can trigger immune-related adverse events (irAEs), among which myocarditis is one of the most life-threatening. Herein, we report the case of a 56-year-old man with HCC who developed myocarditis after receiving two cycles of the PD-1 inhibitor sintilimab. The diagnosis was established via cardiac magnetic resonance (CMR) and confirmed by endomyocardial biopsy (EMB). Following treatment with high-dose corticosteroids and supportive therapy, the patient's condition exhibited significant improvement.

## Introduction

 ICIs targeting programmed death-1/programmed death-ligand 1 (PD-1/PD-L1) and cytotoxic T-lymphocyte–associated protein 4 (CTLA-4) have transformed the landscape of oncology, significantly improving survival outcomes across a wide range of malignancies, including HCC (1, 2). Combination therapies such as atezolizumab plus bevacizumab and nivolumab plus ipilimumab are now established as standard first- or second-line treatments for unresectable HCC (3). Despite these advances, ICIs are associated with a spectrum of irAEs (4). Among these, cardiovascular complications, particularly myocarditis, are relatively rare, occurring in 0.04%–1.14% of patients, yet they carry a high mortality rate of 25%–50% (5, 6). The clinical manifestations of ICI-related myocarditis are highly variable, ranging from asymptomatic elevations in cardiac biomarkers to fulminant heart failure. Therefore, early detection through multimodal evaluation—including cardiac biomarkers, electrocardiography (ECG), echocardiography, and CMR—is crucial for improving patient prognosis (7).

Currently, data on ICI-related myocarditis in patients with HCC remain limited. To address this knowledge gap, we present a case of an HCC patient who developed myocarditis after treatment with sintilimab, as identified by CMR.

## Case presentation

**Figure 1:**
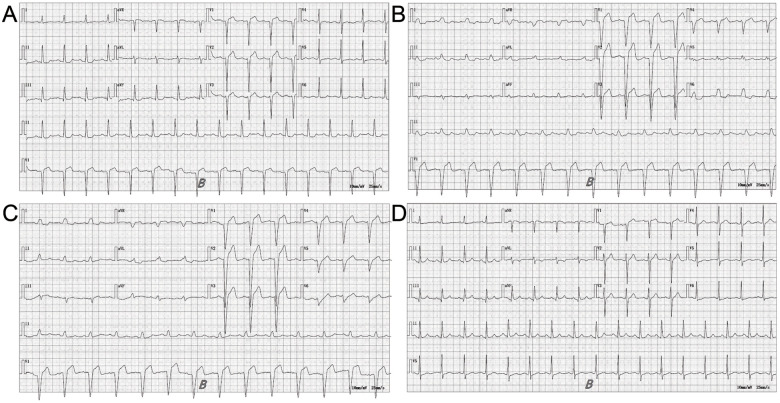
**(A)** Baseline ECG demonstrates nonspecific ST-segment changes, poor R-wave progression in leads V1-V3, a QRS duration of 108 ms, and a QTc interval of 450 ms. **(B)** ECG prior to the second ICI administration reveals a newly developed complete left bundle branch block (CLBBB), persistent nonspecific ST-segment changes, a QS pattern in leads V1-V4, a QRS duration of 152 ms, and a QTc interval of 493 ms. **(C)** ECG at the onset of myocarditis shows an intraventricular conduction delay, with a QRS duration of 167 ms and a QTc interval of 487 ms. **(D)** ECG following high-dose steroid therapy indicates resolution of the conduction abnormalities, with a QRS duration of 97 ms and a QTc interval of 428 ms, though nonspecific ST-segment changes persist.

A 56-year-old man with hepatitis B-related cirrhosis, well-controlled type 2 diabetes, and hypertension was admitted to our institution for a third cycle of PD-1 inhibitor therapy. His treatment course included HCC resection four months prior, followed by the first dose of the PD-1 inhibitor sintilimab (200 mg) one month after surgery. The baseline ECG at therapy initiation (Figure 1A) showed nonspecific ST segment changes and poor R-wave progression in leads V1-V3. Since the patient was asymptomatic following the first cycle, a second cycle was administered two months later. A pre-treatment ECG before the second cycle (Figure 1B), however, showed a newly developed complete left bundle branch block (CLBBB), with a concurrent increase in QRS duration to 152 ms (normal range: 60–100 ms) and a QTc interval of 493 ms (normal range: 300–450 ms).

Twenty days before admission, the patient developed an upper respiratory infection, accompanied by transient chest tightness and dyspnea, although no specific pathogen was identified. Although the symptoms resolved with symptomatic treatment, a coronary angiography (CAG) was performed to evaluate his cardiac complaints, which revealed only mild (40%) stenosis in the proximal segment of the left anterior descending artery.

Upon admission, laboratory tests showed an elevated B-type natriuretic peptide (BNP) level of 117.2 pg/mL(normal range:<100 pg/mL), while high-sensitivity cardiac troponin I (hsTnI) remained within normal limits. The ECG demonstrated an intraventricular conduction block, characterized by a prolonged QRS duration of 167 ms and a QTc interval of 487 ms (Figure 1C). Echocardiography revealed left ventricular (LV) enlargement accompanied by a mildly reduced left ventricular ejection fraction (LVEF) of 45%. Given these abnormalities and the patient's clinical history, CMR was performed on a 1.5 Tesla Philips Ingenia scanner to investigate the underlying etiology of heart failure (HF).

**Figure 2:**
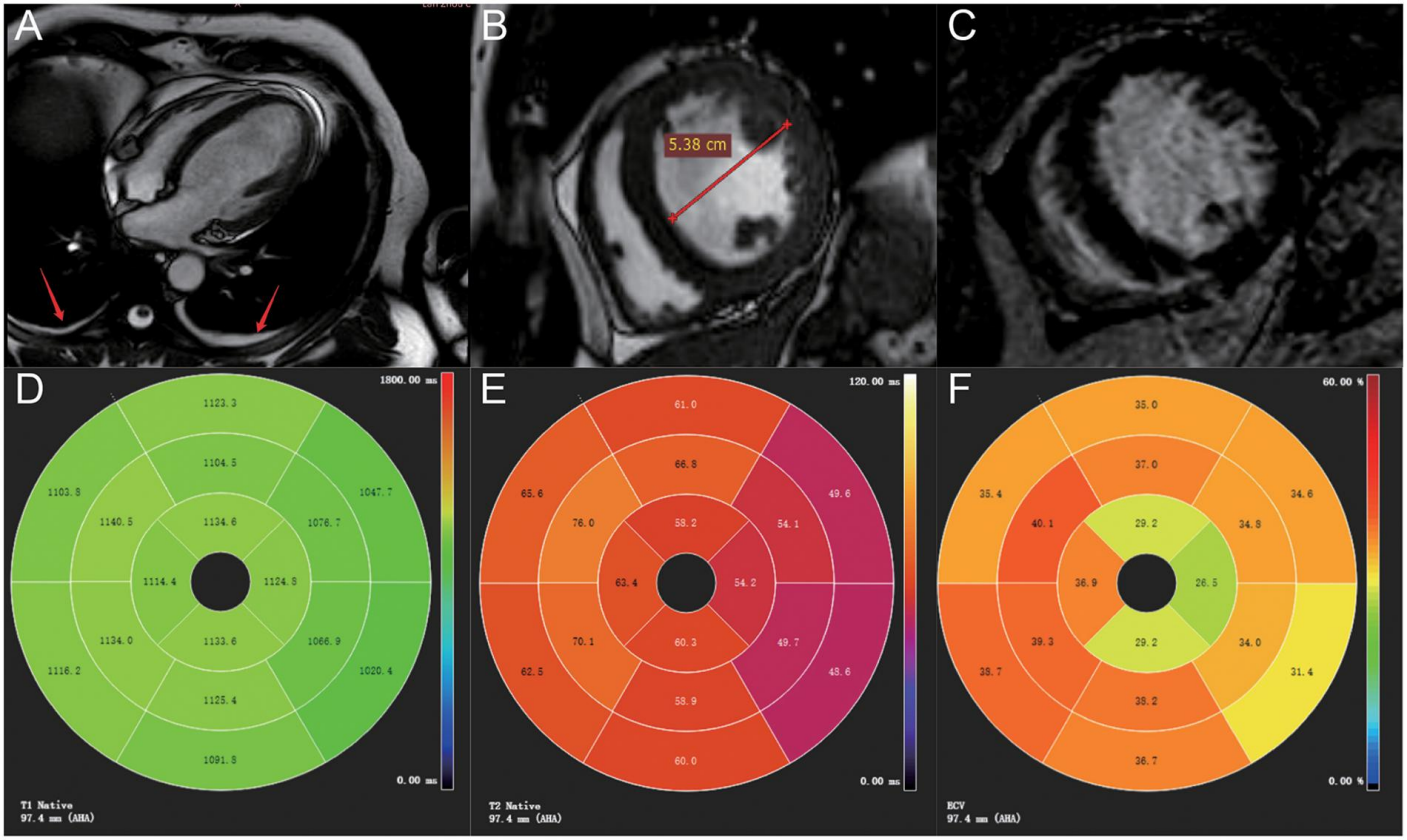
**(A)** Four-chamber cine CMR reveals bilateral pleural effusions (red arrows). **(B)** The short-axis cine CMR measures a left ventricular end-diastolic diameter of 53.8 mm (red line). **(C)** The late gadolinium enhancement image shows no evidence of focal fibrosis. **(D)** T1 mapping demonstrates mildly elevated native T1 values, predominantly in the anterior wall and interventricular septum. **(E)** T2 mapping indicates significantly elevated T2 values, with a similar anterior and septal predominance. **(F)** Extracellular volume map reveals markedly increased values, particularly in the anterior wall and interventricular septum.

The balanced steady-state free precession (bSSFP) cine images acquired in four-chamber and short-axis views (Figures 2A, B) demonstrated a normal LV end-diastolic diameter (53.8 mm), accompanied by septal hypokinesia, mild LV wall thickening, and bilateral symmetrical pleural effusions. T2 mapping (Figure 2E) revealed diffuse myocardial edema, with a T2 value of 60 ms (normal range:＜55 ms). On T1 mapping (Figure 2D) both native T1 values and extracellular volume (ECV) were elevated (T1: 1,098 ms; ECV: 36%; Figure 2F). Late gadolinium enhancement (LGE) images showed no evidence of focal fibrosis (Figure 2C). Based on these CMR characteristics and the patient's history of ICIs therapy, a diagnosis of ICI-related myocarditis was suspected.

**Figure 3:**
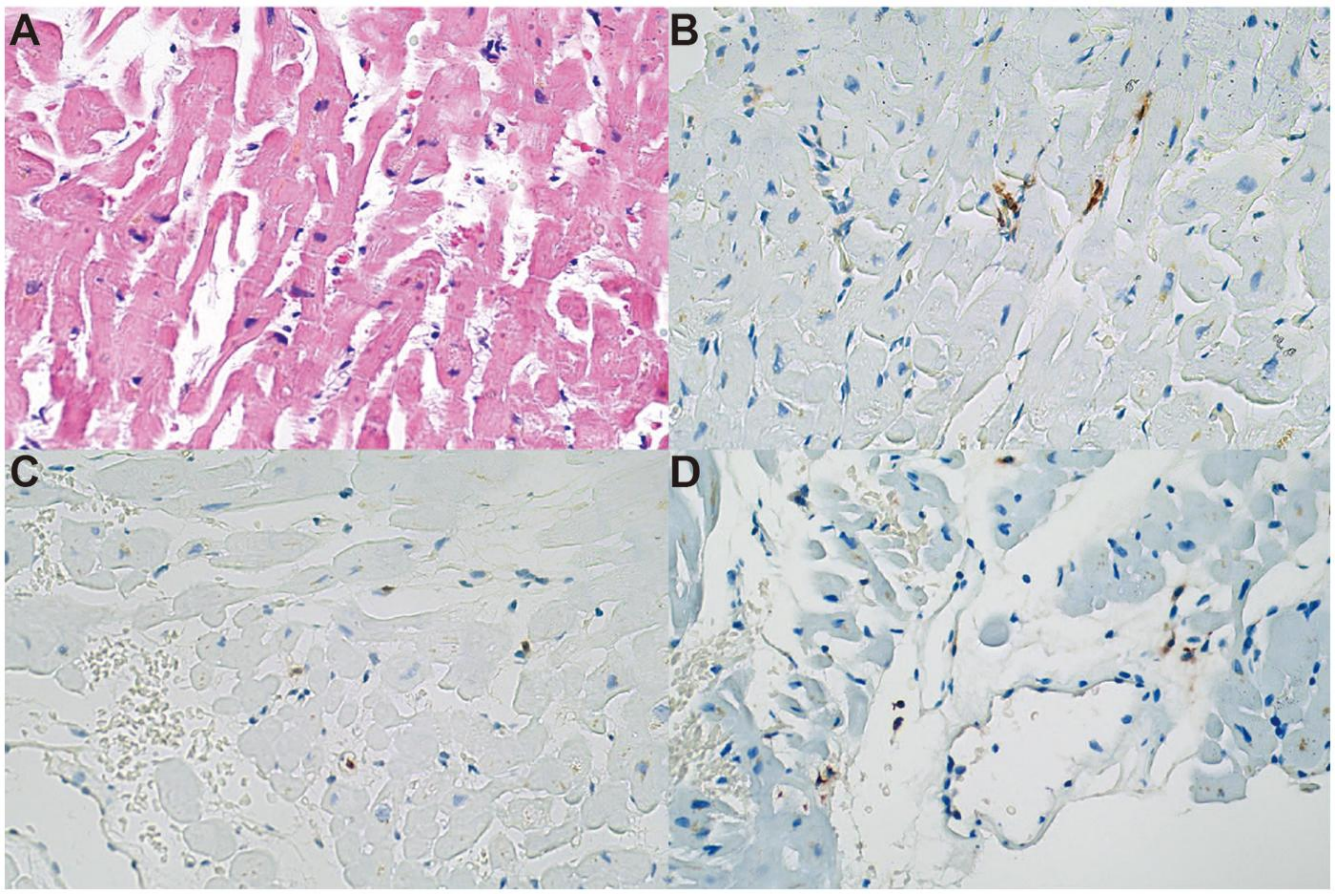
**(A)** Hematoxylin and eosin staining image shows cardiomyocyte hypertrophy, interstitial loosening, edema, and scant lymphocytic infiltration. **(B)** Immunohistochemical staining demonstrates the presence of CD3-positive T lymphocytes. **(C)** Immunohistochemical staining demonstrates the presence of CD4-positive T lymphocytes. **(D)** Immunohistochemical staining demonstrates the presence of CD68-positive macrophages (Magnification:   ×  400).

After exclusion of contraindications, an endomyocardial biopsy (EMB) was performed. Preprocedural laboratory testing revealed an elevated troponin I level (0.069 ng/mL; normal range: 0.010–0.023 ng/mL), with standard N-terminal pro-B-type natriuretic peptide (NT-proBNP) and creatine kinase-MB levels. Histopathological examination of the EMB specimens revealed myocardial fiber hypertrophy, interstitial edema, and sparse lymphocytic infiltration (Figure 3A). Immunohistochemical analysis showed positive staining for CD3⁺/CD4⁺ T cells (Figures 3B,C) and CD68⁺ macrophages (Figure 3D), while staining for CD8⁺ T cells and CD20⁺ B cells was negative. This immunophenotypic profile, characterized by the presence of dominant CD4⁺ T cells and macrophages, supports the diagnosis of ICI-related myocarditis.

Based on the comprehensive clinical presentation, laboratory results, cardiac imaging, and EMB findings, the patient was diagnosed with ICI-related myocarditis. Management was initiated with high-dose intravenous methylprednisolone (1 g/day), followed by a tapered oral regimen. The patient showed significant clinical improvement, with only nonspecific ST-segment changes on ECG (Figure 1D), and was subsequently discharged on a tapering dose of methylprednisolone.

## Discussion

We report the first biopsy-proven sintilimab-associated myocarditis in a patient with resected hepatitis B virus-related HCC, diagnosed by CMR and confirmed by EMB. In this case, the characteristic CD8⁺-predominant inflammatory infiltration typical of viral myocarditis was absent; instead, a CD4⁺/CD68⁺-dominant immune phenotype was observed. Combined with negative pathogen testing and a clear temporal relationship between disease onset and sintilimab infusion, viral myocarditis was reasonably excluded.

The survival of patients with advanced HCC has been markedly enhanced by ICI therapy. The KEYNOTE-224 trial showed a 17% objective response rate for pembrolizumab (8), and the IMbrave150 regimen of atezolizumab plus bevacizumab achieved a median overall survival of 19.2 months (1). Nevertheless, the potential for irAEs must be acknowledged, with ICI-related myocarditis standing out as a notable and severe complication (5).

The pathogenesis of ICI-related myocarditis remains incompletely elucidated. It is primarily driven by T-cell overactivation resulting from the disruption of PD-1/PD-L1 or CTLA-4 signaling pathways, which leads to immune-mediated myocardial injury (9). Other proposed mechanisms include the proliferation of activated T cells and the release of pro-inflammatory cytokines, such as interleukin-2 (IL-2) and interleukin-17 (IL-17) (10). Histologically, we observed CD4⁺-predominant lymphocytic infiltrates with CD68⁺ macrophages—a pattern identical to that reported in pembrolizumab and nivolumab myocarditis, confirming a shared immune mechanism across PD-1 inhibitors.

CD4⁺T cells and CD68⁺macrophages contribute to inflammation by secreting cytokines and amplifying pro-inflammatory responses, whereas CD8⁺T cells primarily mediate direct cytotoxicity against cardiomyocytes. Moreover, the former two cell types are involved in activating and sustaining CD8⁺ T-cell responses.

CD4⁺T cells and CD68⁺macrophages contribute to inflammation by secreting cytokines and amplifying pro-inflammatory responses, whereas CD8⁺T cells primarily mediate direct cytotoxicity against cardiomyocytes. Moreover, the former two cell types are involved in activating and sustaining CD8⁺ T-cell responses.

EMB, while the current diagnostic gold standard, has limited clinical utility due to its invasiveness and sampling variability. Therefore, diagnosis typically relies on a comprehensive approach that incorporates cardiac biomarkers, electrocardiography, and other multimodal imaging modalities.

Notably, with the laboratory test results confirmed to be accurate and reliable, the patient had normal hsTnI levels on admission, whereas troponin I elevation was detected immediately before endomyocardial biopsy. Typical ICI-related myocarditis is generally accompanied by troponin elevation preceding clinical manifestations and imaging abnormalities. This unique phenomenon of delayed biomarker release in this case warrants further investigation.

In the present case, myocarditis developed following the second cycle of sintilimab, aligning with the typical onset pattern reported in the literature (11). This underscores the importance of maintaining a high index of clinical suspicion during the initial cycles of ICI treatment—particularly given that subclinical manifestations may precede overt disease. Notably, new-onset CLBBB appeared before the second infusion. After corticosteroid therapy, CLBBB resolved rapidly, along with prompt normalization of the QRS duration and QTc interval. This suggests that the conduction block was most likely attributable to immune-mediated myocardial inflammation rather than preexisting structural myocardial remodeling, indicating that subclinical myocardial inflammation can be detected by serial ECG. Studies have shown that a QRS duration > 110 ms demonstrates moderate sensitivity (48.6%) and high specificity (87.0%) for diagnosis; when it exceeds 130 ms, sensitivity decreases to 16.4%, while specificity increases to 92.6%. Both QRS and QTc prolongation are associated with an elevated risk of major adverse cardiovascular events (MACE) (12). Consistent with these findings, in the present case, the QRS duration increased from 108 ms prior to ICI therapy to 167 ms during myocarditis, accompanied by QTc prolongation and intraventricular conduction delay. Following ICI discontinuation and corticosteroid treatment, ECG abnormalities improved—further underscoring the value of serial ECG monitoring in the early detection and prognostic evaluation of ICI-related myocarditis.

Twenty days before admission, the patient developed symptoms including chest pain. Although initially attributed to an upper respiratory tract viral infection at a local hospital, these symptoms showed only transient relief with supportive treatment, and no causative pathogen was identified. Following admission, a series of examinations established the diagnosis of ICI-related myocarditis. In retrospect, these symptoms most likely represented early clinical manifestations of ICI-related myocarditis rather than a viral respiratory infection. The symptomatic treatment administered by the local hospital, while temporarily alleviating symptoms, may have inadvertently allowed the underlying condition to progress. Accordingly, in patients undergoing ICI therapy, the onset of symptoms such as chest pain or dyspnea should immediately raise suspicion for ICI-related myocarditis. A comprehensive diagnostic workup is essential for establishing a timely and accurate diagnosis.

CMR is the preferred non-invasive tool for the diagnosis and early detection of ICI-related myocarditis. In this patient, diffuse T2 elevation (60 ms) and an ECV of 36% without LGE reflect reversible edema rather than necrosis. Importantly, an ECV ≥35% is associated with a more than sixfold increased risk of MACE (13); therefore, ECV should always be quantified in suspected cases. Furthermore, when diagnosing ICI-related myocarditis via CMR, it is essential to rule out other myocardial conditions such as ischemic heart disease. In this case, CAG revealed only mild (40%) stenosis, and CMR showed no LGE, thereby excluding an ischemic etiology.

Immediate discontinuation of ICI therapy and prompt initiation of high-dose corticosteroids form the cornerstone of management for ICI-related myocarditis (14). Our patient achieved full ECG recovery within 14 days of 1 g/day methylprednisolone, underscoring the value of early, aggressive immunosuppression.

Although guidelines advise permanent ICI cessation, emerging data suggest that selected low-grade cases may be able to tolerate PD-L1 agents (15). Long-term oncologic follow-up is ongoing to guide potential re-challenge.

The patient remains clinically stable at regular follow-up, with only mild persistent ECG abnormalities indicating a generally good overall condition.

This study has several limitations. First, as it is a single case report, the conclusions cannot be generalized to broader populations. Second, due to the patient's financial constraints, dynamic follow-up evaluation of post-treatment ECV and T2 values was not performed. Third, considering the inherent tissue injury risk of endomyocardial biopsy, only one biopsy was conducted, and no follow-up biopsy was obtained after treatment. Fourth, the patient did not receive regular oncological follow-up for hepatocellular carcinoma after discontinuation of immunotherapy; therefore, the long-term prognosis and therapeutic response of the tumor could not be evaluated.

## Data Availability

The original contributions presented in the study are included in the article/Supplementary Material, further inquiries can be directed to the corresponding author.
